# B7-H3 CAR T cells eradicate intrahepatic cholangiocarcinoma and induce durable response

**DOI:** 10.1186/s13046-026-03723-5

**Published:** 2026-06-04

**Authors:** Shahrzad Arya, Marco Ventin, Giulia Cattaneo, Martina Nebbia, Seyed Amir Sanatkar, Cristina Martin, Jingyu Jia, Bangjin Kim, Cedric A. Bailey, Nabeel M. Bardeesy, David T. Ting, Vikram Deshpande, Xinhui Wang, Sandra Ryeom, Soldano Ferrone, Cristina R. Ferrone

**Affiliations:** 1 Department of Surgery, Division of Gastrointestinal and Surgical Oncology, Massachusetts General Hospital, Harvard Medical School, Boston, MA USA; 2 Department of Surgery, Cedars Sinai Medical Center, Los Angeles, CA USA; 3 Department of Surgery, Herbert Irving Comprehensive Cancer Center, Columbia University Irving Medical Center, New York, NY USA; 4 Department of Pathology, Cedars Sinai Medical Center, Los Angeles, CA USA; 5 MassGeneral Cancer Center, Massachusetts General Hospital, Harvard Medical School, Boston, MA USA; 6 Department of Pathology, Beth Israel Deaconess Medical Center, Boston, MA USA

**Keywords:** B7-H3, CAR T cells, Chimeric antigen receptor, Immunotherapy, Intrahepatic cholangiocarcinoma

## Abstract

**Background:**

Intrahepatic cholangiocarcinoma (ICC) is the second most common primary liver tumor, with an increasing incidence over the past two decades. While surgery offers a curative option, most patients present with advanced disease, for which current therapeutic options are ineffective. B7-H3 is an immune checkpoint molecule that is overexpressed in ICC relative to normal tissue, making it an attractive therapeutic target. Utilizing *in vitro* and *in vivo* models, we investigated the efficacy of a B7-H3-targeted CAR T to treat ICC.

**Methods:**

B7-H3 CAR T cells were generated from peripheral blood mononuclear cells of normal donors, transduced with a retroviral vector encoding a second generation B7-H3 specific CAR construct, incorporating an inducible caspase9 (iCas9) suicide gene-based safety switch. The anti-tumor activity of iCas9.B7-H3 CAR T cells against ICC was tested *in vitro* using human ICC cell lines and patient-derived organotypic tumor spheroids (PDOTS) and *in vivo* using xenograft models of ICC.

**Results:**

Human ICC cell lines, resected ICC samples from patients, and ICC tissue microarrays demonstrate homogeneous expression of B7-H3. iCas9.B7-H3 CAR T cells demonstrated potent anti-tumor activity *in vitro* against multiple patient-derived ICC cell lines. A single systemic dose of iCas9.B7-H3 CAR T cells induced complete and sustained eradication of orthotopic ICC tumors in a preclinical mouse model, even after tumor re-challenge. Locoregional delivery of CAR T cells via splenic and intra-tumoral injection was equally effective as systemic therapy.

**Conclusions:**

These results provide a strong rationale for evaluating the iCas9.B7-H3 CAR T cell strategy in clinical trials for patients with advanced ICC.

**Supplementary Information:**

The online version contains supplementary material available at https://doi.org/10.1186/s13046-026-03723-5.

## Background

The incidence of intrahepatic cholangiocarcinoma (ICC) continues to rise, making it the second most common primary hepatic cancer, following hepatocellular carcinoma [1–3]. Surgical resection provides the only curative treatment option, but [4, 5] more than half of patients present with unresectable disease due to locally advanced or metastatic tumors [6, 7]. At this stage, therapeutic options have limited efficacy, with chemotherapy (gemcitabine/cisplatin) and anti-PD-L1 immunotherapy (durvalumab) as the current front-line systemic therapy. Despite these treatments, long-term outcomes remain poor, with 5-year survival rates of only 20–35% [8, 9].

High rates of tumor recurrence further contribute to poor prognosis, with 50% to 70% of patients developing local or distant relapse involving liver, lungs, lymph nodes, peritoneum, or bones [10, 11]. Although additional therapies such as chemotherapy, radiotherapy, hepatic artery (HA)-based therapies, and, more recently, immunotherapy are available, their clinical benefit remains limited, and most patients with ICC will ultimately succumb to metastatic disease [7, 8, 12]. These limitations underscore the need for more effective therapeutic strategies for advanced ICC with T-cell-based immunotherapy approaches at the forefront of preclinical and clinical investigations for patients with advanced ICC [13, 14].

Previous studies have demonstrated that some biliary tract cancers, including ICC, have a higher tumor mutation burden (TMB) and PD-L1 expression, suggesting that a subset of patients may benefit from immune checkpoint inhibitors [15–17]. However, defects in HLA class I antigen presentation are common in ICC, enabling tumor cells to evade cytotoxic T-cell recognition. These findings highlight the need for immunotherapeutic approaches that bypass HLA-restricted antigen presentation. Chimeric antigen receptor (CAR) T-cell therapy enables HLA Class I-independent recognition of tumor-specific antigens [18] and has demonstrated remarkable efficacy in several hematologic malignancies [19–22]. The use of CAR T therapies against solid tumors has promising results in several clinical trials [23–25].

B7-H3 (CD276) is a type I transmembrane protein and part of the B7-ligand family of immunomodulatory molecules. It has emerged as a promising therapeutic target in several solid tumors, including ICC. B7-H3 is frequently overexpressed on malignant cells and has been associated with ICC tumor invasion, metastases, and poor patient prognosis [26]. Importantly, its expression in normal tissues is limited [27–30], suggesting a potential therapeutic window for B7-H3 targeted therapies.

Prior studies demonstrate increased expression of B7-H3 in ICC with B7-H3 expression reported in approximately 50–60% of ICC tumors, with minimal or no expression in non-neoplastic bile duct tissue. Moreover, B7-H3 expression has been associated with adverse clinicopathologic features, including lymph node metastasis, venous invasion, and reduced survival [26]. In addition to malignant epithelial cells, B7-H3 has also been detected in tumor-associated stroma and vasculature, including cancer-associated fibroblasts, suggesting that targeting this antigen may also modulate the tumor microenvironment and tumor angiogenesis [31–34]. These findings support B7-H3 as a biologically relevant and potentially actionable target in ICC.

In this study, we evaluated the therapeutic potential of B7-H3–directed CAR T cells incorporating an inducible caspase-9 safety switch in preclinical models of ICC. We demonstrate potent antitumor activity, dose-dependent tumor control, and durable responses following tumor rechallenge in orthotopic xenograft models, supporting the translational potential of B7-H3 CAR T therapy for advanced ICC.

## Methods

### Study approval

All mouse studies were performed under protocols (#2018N000208 and #2021N000244) approved by the Institutional Animal Care and Use Committee (IACUC) at the Massachusetts General Hospital. Patient tissue sample collection was performed under a protocol (#02–240) approved by the Institutional Review Board (IRB) at Dana-Farber/Harvard Cancer Center.

### Cell lines and culture

Human intrahepatic cholangiocarcinoma (ICC) cell lines (ICC2, ICC3, ICC20, ICC21, ICC137) were generated by Dr. Cristina R. Ferrone, Dr. Nabeel Bardeesy, and Dr. Matteo Ligorio, as per our Institutional Review Board approved protocol (DFCI, #13–162, #13–416, and 19–699) at the Massachusetts General Hospital and are not commercially available [35]. Established cell lines were obtained from the following sources: Riken Bioresource Center (RBE) and Korean Cell Line Bank (SNU1079). The ICC cell lines ICC2 and ICC3 were stably transduced with a lentiviral vector encoding Green Fluorescent Protein (GFP) and luciferase (Luc) and are named ICC2-GFP.Luc and ICC3-GFP.Luc. Lentiviral supernatant for transduction was produced by transfecting HEK293T cells with the pMD2.G plasmid (envelope), the psPAX2 plasmid (gag-pol), and the lentiviral vector encoding the GFP.Luc construct (kindly provided by Dr. Joseph Franses, Massachusetts General Hospital). All ICC cell lines were cultured in RPMI-1640 (Corning) culture medium supplemented with 10% FBS (Gemini Bio) and 2 mM L-glutamine (ThermoFisher) in a humidified 5% CO2 incubator at 37 °C. Jurkat cells were purchased from the American Type Culture Collection (ATCC) and cultured in RPMI-1640 supplemented with 10% FBS. Routine testing for Mycoplasma contamination was performed using the MycoAlert® Mycoplasma detection kit (Lonza).

### Human ICC and normal tissue samples

Human ICC tissue samples were obtained from surgically removed primary and metastatic lesions of ICC patients under an IRB-approved protocol (#02–240). In addition, a commercially available cholangiocarcinoma tissue microarray (TMA; product #GA802b; TissueArray) containing intrahepatic and extrahepatic cholangiocarcinoma specimens, along with normal adjacent liver tissue, was used for immunohistochemical analysis.

### Immunohistochemistry

Fresh murine ICC specimens were formalin-fixed and paraffin-embedded by the Histology Research Core Facility at MGH, cut into 4 μm thick sections on Superfrost Plus slides (12–550-15, Fisher Scientific). Immunohistochemistry (IHC) staining was automatically performed on the Ventana Discovery Ultra Platform. After antigen retrieval CC1 (pH8.0) (950–124, Roche) for CD3 and CC2 (pH6.0) (950–123, Roche) for CD276, two primary antibodies were applied individually. CD276 was diluted with an Antibody Dilution Buffer (ADB250, Roche). Anti-CD3 (790–4341, Roche) prediluted and prefilled, incubated 32 min at 37 °C; 2) and anti-CD276 (ab227670, abcam) 1:400, incubated 1 h at Room Temperature. Then following the detection system of DISC anti-Rabbit HQ (760–4815, Roche) and DISC anti-HQ HRP (760–4820, Roche). DISC ChromoMap DAB Kit (760–159, Roche) was finally applied. Stained slides were digitally imaged using the Aperio ScanScope XT (Leica).

### Tumor necrosis assessment

Tumor necrosis was evaluated on hematoxylin and eosin–stained sections using a standardized histopathologic scoring approach performed by a board-certified surgical pathologist. Necrosis was defined as areas of non-viable tumor characterized by loss of cellular architecture and increased eosinophilia, and was quantified relative to the total tumor area.

### Tissue microarray (TMA) analysis and scoring

TMA sections were stained for B7-H3 as described above. Whole-slide images were digitally scanned and analyzed using QuPath software (version 0.7.0) for automated quantification. Tumor regions were annotated, and positive tumor cells were detected using color deconvolution–based DAB detection. B7-H3 expression was quantified as the percentage of B7-H3–positive cells and by H-score, calculated from the proportion of cells with weak, moderate, and strong staining intensities detected by the software using the formula: H-score = (1 × % weak) + (2 × % moderate) + (3 × % strong), yielding a score ranging from 0 to 300. Each data point in the quantitative plots represents an individual TMA core.

### Generation of CAR T cells

#### Retroviral supernatant preparation

The variable regions of the heavy and light chains from the 376.96 mouse hybridoma were cloned to create a single-chain variable fragment (scFv) [36]. This scFv fragment was integrated into a CAR cassette incorporating a human CD8a hinge and transmembrane domain: a CD28 intracellular costimulatory domain and a CD3ζ intracellular signaling domain. The resulting B7-H3 CAR cassette was then inserted into the retroviral vector SFG. Retroviral supernatants used for transducing human T cells were generated by transfecting HEK293T cells with the RDF plasmid encoding the RD114 envelope, the PegPam3 plasmid (gag-pol), and the retroviral vector containing the iCas9.B7-H3-28ζ CAR, B7-H3-28ζ CAR, or CD19-28ζ CAR construct (kindly provided by Dr. Gianpietro Dotti, University of North Carolina at Chapel Hill, Chapel Hill, NC, USA), as previously described. The RDF and PegPam3 plasmids encode essential components for retroviral production [37].

#### Transduction and expansion of CAR T cells

Second-generation CAR T cells were generated from unpurified buffy coats from normal human adult donors (Research Blood Components). Peripheral blood mononuclear cells (PBMC) were isolated by density gradient centrifugation (Lymphoprep™, STEMCELL, 07861) and plated in a 24-well non-treated tissue culture plate (ThermoFisher) pre-coated with CD3 (1 μg/ml, Miltenyi, Bio130-093–387) and CD28 (1 μg/ml, BD Bioscience, 556,620) antibodies to induce T cell activation. The culture medium consisted of equal parts Click’s Medium (Irvine Scientific, 9195) and RPMI-1640 (Corning, 10–040-CM) supplemented with 10% FBS (Gemini Bio, S12450), penicillin (100 U/ml)/streptomycin (100 U/ml), L-glutamine (2 mM, ThermoFisher, 25,030,081), IL-7 (10 ng/ml, Peprotech, 200–07), and IL-15 (5 ng/ml, Peprotech, 200–15). Non-tissue culture-treated 24-well plates were coated overnight with 7 mg/mL retronectin (Takara Bio, T100B) at 4 °C, washed once with 1 mL culture medium, coated with 1 mL/well of retroviral supernatant, and centrifuged at 2000 g for 90 min. After removing the supernatant, 4 × 10^5^ activated T cells were plated and centrifuged at 1000 g for 10 min. After 72 h, the viral supernatant was removed and replaced with fresh culture medium supplemented with IL-7/IL-15 to allow CAR T cell expansion.

### *In vitro *co-culture experiments and cytotoxicity assays

#### 3-(4,5-dimethylthiazol-2-yl)−2,5-diphenyl-2H-tetrazolium bromide (MTT) assay

Tumor cells were seeded in 96-well tissue culture plates (2500 cells/well). iCas9.B7-H3 CAR T cells were added to the culture at effector (CAR T) to target (cancer cells) (E:T) ratios of 1:1, 1:5, 1:10, and 1:20 and incubated at 37 °C, 5% CO2 without the addition of exogenous cytokines. Following a 72-h incubation, CAR T cells were removed by washing each well twice with 200 μL PBS, and tumor cell adherence was visually confirmed prior to the addition of MTT reagent, after which, cancer cell viability was assessed by the MTT assay (Millipore Sigma) according to the manufacturer’s instructions. Control groups, including untreated cancer cells, were included for baseline comparison.

#### Live cell-imaging

ICC3-GFP.Luc cell line was seeded in 96-well tissue culture plates (2500 cells/well). iCas9.B7-H3 CAR T cells were added to the culture at E:T ratios of 1:1, 1:5, and 1:20 without the addition of exogenous cytokines. Tumor cell proliferation was continuously monitored for 96 h using an Incucyte® SX5 Live-Cell Analysis Instrument.

#### Spheroid preparation and microfluidic culture

Patient-derived organotypic tumor spheroids (PDOTS) were created and cultured following established methods [38]. A 10 µL mixture of spheroids and collagen was placed in the central gel area of a 3D microfluidic culture device (AIM Dax-01, AIM Biotech). The collagen hydrogels containing the tumor spheroids were then incubated for 30 min at 37 °C in sterile humidity chambers. Post-incubation, the hydrogels were hydrated with a culture medium (DMEM with 10% FBS and 1% penicillin–streptomycin). CAR T cells were introduced into the side channel of the culture device at an E:T ratio of 1:1. To evaluate the viability of the PDOTS, we utilized a dual-label fluorescence live/dead staining method along with Hoechst dye (Sigma-Aldrich, 23,491–45-4) to label nuclei and Propidium Iodide (Invitrogen, bMS500PI) [38]. After a 45-min incubation with the Ho/PI at 37 °C and 5% CO2, images were captured using a Nikon Eclipse NiE fluorescence microscope with a motorized stage (ProScan) and a ZYLA4.2 Plus USB3 Camera (Andor), along with the NIS-Elements AR software. CAR T cells were labeled with CFSE (Invitrogen, C34554) prior to co-culture to enable visualization of T-cell localization within spheroids. The quantification of live and dead cells was done by measuring the total raw cell area for each dye. Changes in viability and log2 fold change (L2FC) data were then calculated based on raw fluorescence data (live) for each treatment compared to control conditions.

### Effector cytokine profiling assay

Tumor cells were seeded in 24-well tissue culture plates (50,000 cells/well). iCas9.B7-H3 CAR T cells were added to the culture at the E:T ratio of 1:1 and incubated at 37 °C without the addition of exogenous cytokines. Following a 24-h incubation period, cell culture supernatants were collected, and CD8 T/NK cell effector cytokines produced by iCas9.B7-H3 CAR T cells were measured utilizing a bead-based immunoassay (LEGENDplex™ V-Bottom Multiplex Assay kit for Human CD8/NK Panel, 741,065) following the manufacturer’s instructions.

### Flow cytometry

For *in vitro* co-culture experiments, ICC-GFP.Luc cell lines were seeded in 6-well plates (2 × 10^5^ cells/well) and co-cultured with iCas9.B7-H3 CAR T cells at an effector-to-target (E:T) ratio of 1:1 without exogenous cytokines. After 48 h, cells were harvested and stained. Dead cells were excluded using Propidium Iodide (Invitrogen, bMS500PI). CAR T cells were identified by CD3 expression (BioLegend, 300,405, clone OKT3), and tumor cells by GFP expression. Absolute numbers of viable tumor cells were determined using CountBright™ Absolute Counting Beads (Thermo Fisher, C36950). Unstained and single-stained controls were used for compensation and gating. For PDOT immune profiling, spheroids were dissociated into single-cell suspensions and stained with antibodies against EpCAM (BioLegend, 324,235, clone 9C4), CD3 (BioLegend, 300,405, clone OKT3), CD33 (BioLegend, 303,407, clone WM53), and CD45 (Bio-Rad, MCA87SBUV445, clone F10-89–4). Cell populations were identified by sequential gating and expressed as percentages of total viable cells. For analysis of CD19 expression on ICC cells, APC anti-human CD19 antibody (BioLegend, 302,211, clone HIB19) was used. For analysis of B7-H3 expression, fresh tumor and matched normal tissues were enzymatically digested using collagenase IV (0.5 mg/mL; Sigma-Aldrich) for 2 h at 37 °C and filtered through 70 μm strainers to obtain single-cell suspensions. Red blood cells were lysed using ACK Lysing Buffer (Thermo Fisher, A1049201), and viability staining was performed using Propidium Iodide. B7-H3 expression was assessed using the 376.96 mouse anti-human B7-H3 monoclonal antibody (1 μg/mL), followed by APC-conjugated goat anti-mouse secondary antibody (Jackson ImmunoResearch). CAR T-cell transduction efficiency and CAR expression were evaluated using FITC-labeled human B7-H3 protein (Acro Biosystems, B7B-HF2E7) and CD19-based detection reagents recognizing the CAR construct, including FITC-labeled human CD19 protein (Acro Biosystems, CD9-HF2H2-25ug). Phenotypic characterization was performed using antibodies against CD3 (BioLegend, 300,420, clone UCHT1), CD4 (BioLegend, 300,506, clone RPA-T4), CD8 (BioLegend, 344,705, clone SK1), CD45 (BioLegend, 368,510, clone 2D1), CD45RO (BioLegend, 304,210, clone UCHL1), CD62L (BioLegend, 304,804, clone DREG-56), PD-1 (BioLegend, 367,410, clone NAT105), LAG-3 (BioLegend, 369,212, clone 7H2C65), TIM-3 (BioLegend, 345,022, clone F38-2E2), and CD69 (BioLegend, 310,930, clone FN50). ALDH activity was assessed using the ALDEFLUOR kit (STEMCELL Technologies, 01700). Where indicated, CAR T cells were treated with rimiducid (AP1903; MedChemExpress, HY-16046). All samples were acquired using BD FACSDiva (v8.0) or a SONY ID7000 Spectral Cell Analyzer and analyzed using FlowJo v10.9.

### *In vivo* mouse studies

#### Ethics statement

All animal studies were conducted in compliance with protocols approved by the Institutional Animal Care and Use Committees (IACUC) at both Massachusetts General Hospital (MGH) and Cedars-Sinai Medical Center. Experiments were performed in accordance with institutional guidelines and all relevant regulations governing the ethical treatment of animals. The maximal tumor burden permitted by the IACUC protocols at both institutions was 1,500 mm^3^. At no time during the course of the experiments did tumor size exceed this threshold. Animals were monitored daily for tumor progression and overall health, and humane endpoints were strictly observed. Source data supporting all figures describing tumor growth are available by the journal’s data availability policy.

#### Mice

Eight-week-old female and male NOD.Cg-Prkdcscid Il2rgtm1Wjl/SzJ (NSG, Strain #005557) mice were purchased from The Jackson Laboratory. All mice were housed, and experiments were performed in the Animal Facility at MGH. Animal studies were carried out according to the ethical regulations and protocols under the approval of the MGH Institutional Animal Care and Use Committee (IACUC).

#### ICC orthotopic models

ICC3-GFP.Luc tumor cells (5 × 10^5^ cells/mouse) were suspended in 20 μl FBS-free RPMI medium and engrafted in the liver of 8-week-old NSG mice (male and female). Mice were anesthetized with ketamine (80–100 mg/kg)/xylazine (5–10 mg/kg) and placed in a supine position. After disinfecting the skin, a 1 cm left midline incision exposed the liver, onto which tumor cells were injected using a 31G sterile syringe. Surgifoam (Ethicon) (0.5 × 0.5 cm^2^) was placed at the injection site to prevent bleeding and cell leakage. The wound was closed in two layers using 4–0 monocryl and 4–0 Ethicon sutures, and a stapler (9 mm Autoclip applier). Seven days post-engraftment, iCas9.B7-H3-28ζ CAR T cells or CD19-28ζ CAR T cells (5 × 10^6^ CAR T cells/mouse) were injected intravenously (i.v.). Thirty days later, mice were re-challenged by i.v. injection of 2.5 × 10^5^ ICC3-GFP.Luc cells and monitored for 20 weeks.

For the CAR T route of administration experiment, in splenic injection, mice were anesthetized with ketamine/xylazine, and iCas9.B7-H3 CAR T cells (5 × 10^6^ cells/mouse) were injected into the spleen. After hemostasis, splenic vessels were ligated, and a splenectomy was performed. For intra-tumoral injection, mice were anesthetized, and iCas9.B7-H3 CAR T cells (5 × 10^6^ cells/mouse) were injected adjacent to and into the tumor. Incisions were closed as previously described.

### In vivo imaging

#### Bioluminescence imaging

Mice were anesthetized using ~ 2% isoflurane in oxygen and intraperitoneally injected with D-luciferin Firefly (MedChemExpress, HY-12591A). After a specified time post-injection (e.g., 5 min), fluorescence intensity was measured using the AMI HTX *in vivo* imaging system (Spectral Instruments Imaging). Image processing and the quantification of photon flux were performed with Aura Imaging software (v. 4.0).

### Statistical methods, data analysis, and software

Unless otherwise indicated, all graphs with error bars represent mean ± standard deviation (SD) values. Statistical analyses were conducted using specific tests, including [t-tests, two-way ANOVA, and log-rank tests]. The number of replicates and independent experiments is detailed in the figure legends. GraphPad Prism v 10 (GraphPad, La Jolla, CA) was utilized for basic statistical analysis, graph plotting, and visualization. The publicly available TCGA cholangiocarcinoma dataset (TCGA-CHOL) was utilized to evaluate B7-H3 (CD276) mRNA expression. Analysis was performed using GEPIA2. Protein-level expression of B7-H3 (CD276) in normal and malignant biliary tissues and hepatobiliary cancer cell lines was assessed using the Human Protein Atlas.

## Results

### B7-H3 expression on ICC cell lines and tumors

**Fig. 1:**
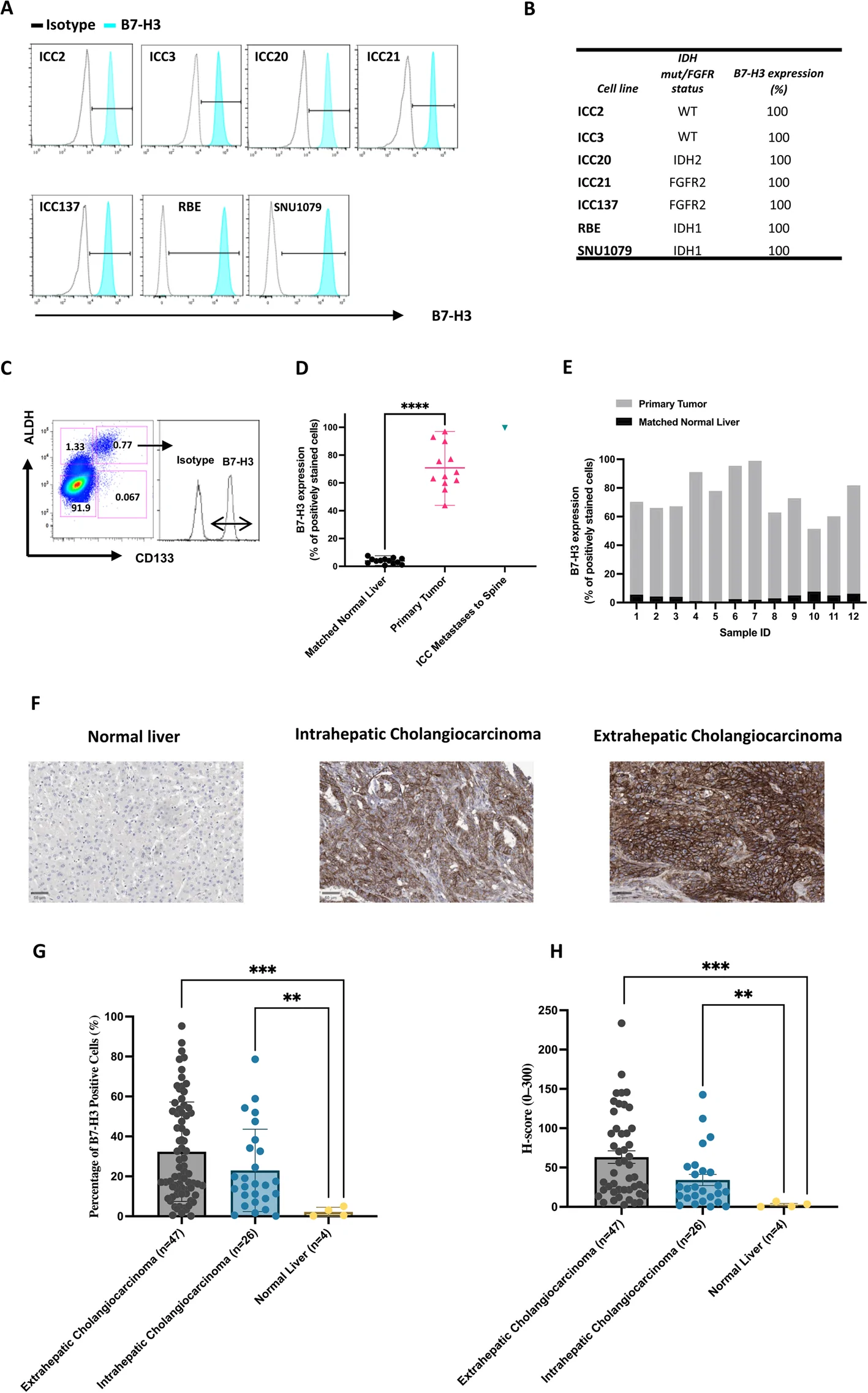
B7-H3 is overexpressed in primary and metastatic ICC, including cancer-initiating cells. **A**,** B** Representative flow cytometry histograms demonstrating surface B7-H3 expression in multiple patient-derived and commercially available ICC cell lines stained with anti–human B7-H3 mAb (376.96, 1 μg/mL). **C** Identification of ICC cancer-initiating cells (CICs) based on ALDH and CD133 expression (left). ALDH⁺CD133⁺ cells demonstrate high B7-H3 surface expression (right). **D** Quantification of B7-H3 expression by flow cytometry in human ICC tumor samples (*n* = 13) and matched normal liver tissues (*n* = 13). **E** Representative flow cytometry histograms comparing B7-H3 expression in tumor versus matched normal liver tissue. **F** Representative B7-H3 immunohistochemistry (IHC) images from a tissue microarray (TMA) containing normal liver, extrahepatic and intrahepatic cholangiocarcinoma (scale bar, 50 μm). **G** Quantification of B7-H3–positive cells in TMA sections including extrahepatic cholangiocarcinoma (*n* = 47), intrahepatic cholangiocarcinoma (*n* = 26), and normal adjacent liver tissue (*n* = 4). **H** H-score quantification of B7-H3 expression across the TMA. Each dot represents an individual TMA core. Statistical analysis: Paired comparisons were performed using paired t-tests (**D**); group comparisons were performed using the Mann–Whitney U test **(G**, **H**). **P ≤ 0.01; ***P ≤ 0.001; ****P ≤ 0.0001

To evaluate B7-H3 expression on human ICC cell lines, we performed flow cytometry analysis. Our results demonstrate uniform and high membrane expression of B7-H3 in seven different patient-derived and commercially available ICC cell lines (ICC2, ICC3, ICC20, ICC21, ICC137, RBE, and SNU1079) (Fig. 1A, 1B) that all lacked CD19 expression (Fig. S1A). ICC3 was selected for subsequent functional and *in vivo* studies due to its consistent B7-H3 expression and reproducible tumor growth in preclinical orthotopic models. We investigated B7-H3 expression in cancer-initiating cells (CICs) as this population has been implicated in chemoresistance and disease recurrence. CICs were identified based on the well-established markers, aldehyde dehydrogenase (ALDH) activity and CD133 expression [39, 40]. B7-H3 expression was also expressed in this CIC subpopulation, as indicated by co-expression of ALDH, CD133, and B7-H3 in the ICC3 cell line (Fig. 1C). We next assessed B7-H3 expression in primary ICC patient specimens. B7-H3 expression was confirmed in surgically resected primary and metastatic ICC tumors by flow cytometric analysis of single-cell suspensions (Fig. 1D). Among the 13 unique patient specimens examined, 92% demonstrated 50–100% B7-H3 staining (*n* = 12/13), while 8% of tumors exhibited 40–50% positively stained cells (*n* = 1/13) (Table S1). In contrast, matched normal human liver tissues demonstrated minimal to no B7-H3 expression. B7-H3 expression was significantly higher in tumor samples as compared to adjacent normal liver tissues (Fig. 1E). To further validate B7-H3 expression in ICC patient samples, we analyzed a cholangiocarcinoma tissue microarray containing extrahepatic biliary duct adenocarcinoma (*n* = 47), intrahepatic cholangiocarcinoma (*n* = 26), and normal adjacent liver tissues (*n* = 4). Immunohistochemical staining demonstrated extensive B7-H3 expression in tumor specimens, whereas minimal staining was observed in normal liver tissue (Fig. 1F). Quantification of B7-H3 expression either by the percentage of B7-H3 positive cells (Fig. 1G) or by H-score (Fig. 1H), confirmed significant upregulation of B7-H3 in cholangiocarcinoma as compared to normal liver samples. We interrogated the TCGA-cholangiogcarcinoma (CHOL) cohort using GEPIA2 for B7-H3 mRNA expression and found significantly higher *CD276* (B7-H3) mRNA expression in ICC tumors (*n* = 36) compared to normal hepatobiliary tissues (*n* = 9) (Fig. S2A). Survival analysis showed an association between high B7-H3 expression and decreased overall survival (HR = 1.4, log-rank *p* = 0.48), although this did not reach statistical significance (Fig. S2B). In addition, Human Protein Atlas datasets demonstrated heterogeneous but low CD276 expression across normal hepatobiliary cell populations, including cholangiocytes and hepatocytes, whereas cholangiocarcinoma cell lines consistently exhibited higher expression levels (Fig. S2C–D).

### *In vitro* anti-tumor activity of B7-H3 CAR T cells

**Fig. 2:**
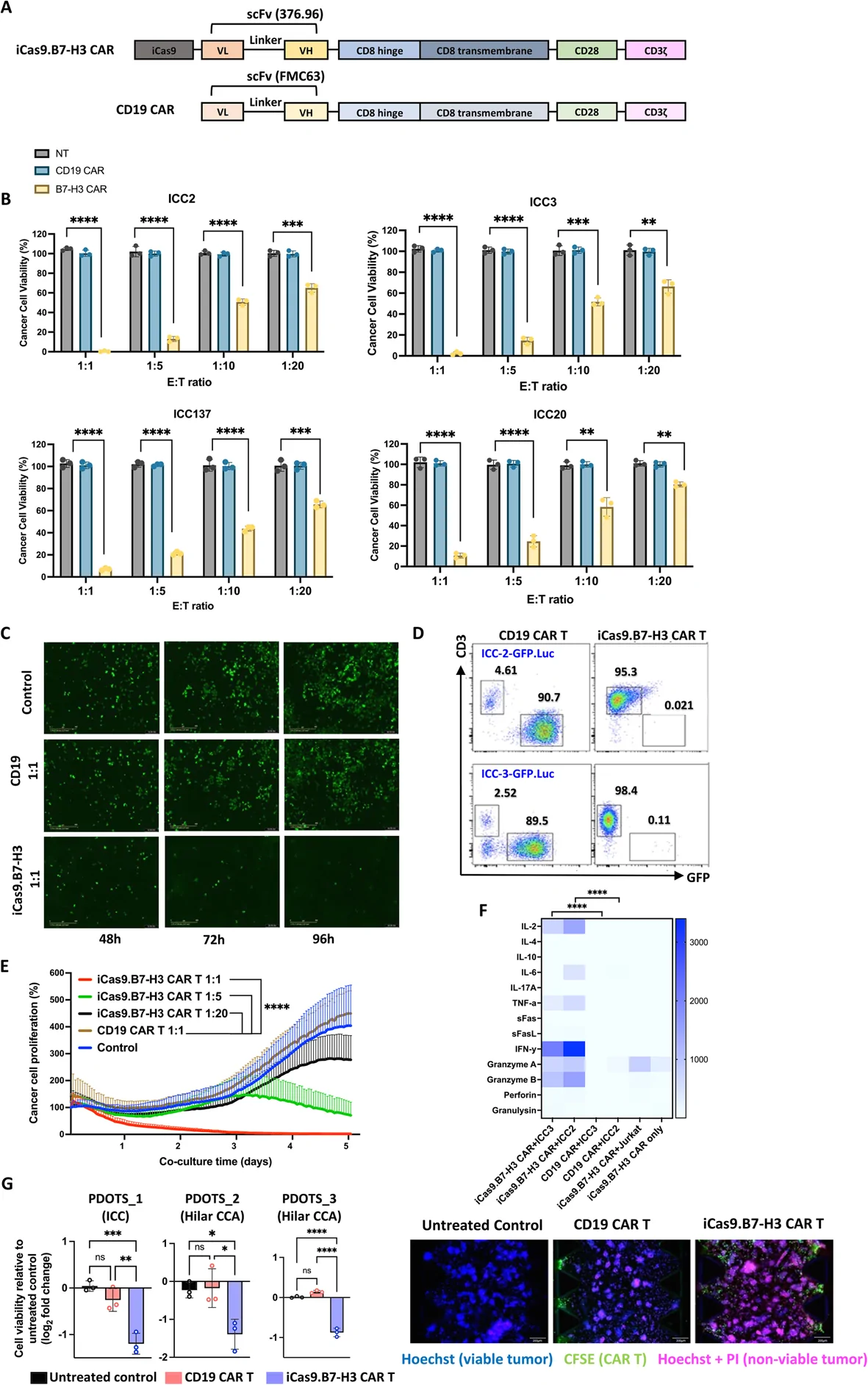
*In vitro* activity of iCas9.B7-H3 CAR T cells against ICC. **A** Schematic representation of the iCas9.B7-H3 CAR construct incorporating an inducible caspase-9 (iCas9) safety switch, and the control CD19 CAR construct. **B** Quantification of ICC cell viability following co-culture with iCas9.B7-H3 CAR T cells, CD19 CAR T cells, or non-transduced (NT) T cells at the indicated effector-to-target (E:T) ratios, assessed by MTT assay after 72 h (*n* = 3 independent experiments). **C** Representative fluorescence microscopy images showing reduction of GFP⁺ ICC3 tumor cells following co-culture with iCas9.B7-H3 or CD19 CAR T cells at a 1:1 E:T ratio over time (48–96 h) (scale bar, 400 μm). **D** Representative flow cytometry plots demonstrating residual tumor cells after 48 h of co-culture with iCas9.B7-H3 or CD19 CAR T cells at a 1:1 E:T ratio. **E** Real-time monitoring of ICC3 tumor cell growth during co-culture with iCas9.B7-H3 CAR T cells over 5 days. **F** Quantification of effector cytokines released by iCas9.B7-H3 or CD19 CAR T cells in culture supernatants after 24 h of co-culture with indicated ICC cell lines (*n* = 3 independent experiments). **G** Viability assessment of patient-derived organotypic tumor spheroids (PDOTS) from intrahepatic and extrahepatic cholangiocarcinoma following 5 days of co-culture with iCas9.B7-H3 or CD19 CAR T cells in a 3D microfluidic system (*n* = 3 technical replicates). Quantification of cell viability and representative images of PDOTS with the indicated treatments (scale bar, 200 μm). Hoechst (blue) labels viable tumor cells, CFSE (green) labels CAR T cells, and propidium iodide (magenta) identifies non-viable tumor cells. Statistical analysis: Multiple unpaired t-tests (B), paired t-test (E), two-way ANOVA with Geisser–Greenhouse correction (F), and one-way ANOVA (G). *P ≤ 0.05; **P ≤ 0.01; ***P ≤ 0.001; ****P ≤ 0.0001

We recently published the generation of a B7-H3-targeted CAR T cell with an inducible caspase-9-dependent suicide gene (iCas9.B7-H3) [41], and utilized this CAR T as well as a CD19 CAR T as a negative control (Fig. 2A). The incorporation of the inducible caspase-9 (iCas9) suicide gene did not compromise the transduction efficiency or effector function of B7-H3-directed CAR T cells. Both B7-H3 CAR T and iCas9.B7-H3 CAR T cells, generated from three independent healthy donors, exhibited comparable transduction efficiencies (B7-H3 CAR: 85.9 ± 0.5% vs. iCas9.B7-H3 CAR: 84.9 ± 2.0%, p = 0.522) (Fig. S3A). In co-culture assays with ICC3 tumor cells, both CAR T cells demonstrated similar levels of antitumor activity across various effector-to-target (E:T) ratios, indicating that the presence of the iCas9 construct did not impair cytotoxic function (Fig. S3B). Administration of the chemical inducer of dimerization (AP1903) effectively triggered rapid and selective *in vitro* elimination of iCas9.B7-H3 CAR T cells, confirming the functionality of the iCas9-based safety switch (Fig. S3C).

We next evaluated the anti-tumor activity of iCas9.B7-H3 CAR T cells against human ICC cell lines with different genetic backgrounds, including wild type (ICC2 and ICC3), IDH2 mutated (ICC20), and FGFR2 fusion (ICC137). CD19 CAR T cells and non-transduced T cells were used as negative controls. iCas9.B7-H3 CAR T cells effectively eliminated ICC cells at an E:T ratio of 1:1 and demonstrated dose-dependent anti-tumor activity at lower E:T ratios. In comparison, CD19 CAR T and non-transduced T cells had no effect on ICC cells, indicating the specificity of our iCas9.B7-H3 CAR T cells (Fig. 2B-C). To assess antigen-driven proliferation of our CAR T, equal numbers of iCas9.B7-H3 and CD19 CAR T cells were co-cultured with ICC2 and ICC3, at a 1:1 E:T ratio for 3 days. Notably, iCas9.B7-H3 CAR T cells exhibited robust proliferation in response to ICC cells, whereas CD19 CAR T cells did not expand (Fig. 2D).

To further validate the cytotoxicity observed in Fig. 2B–C, we performed longitudinal live-cell imaging of CAR T-cell co-cultures with ICC3 cells. Over five days, iCas9.B7-H3 CAR T cells progressively eradicated ICC3-GFP.Luc cells (Fig. 2C, 2E). These findings confirm that tumor elimination is sustained over time and dependent on antigen-specific CAR T-cell activity. Consistent with these observations, co-culture with ICC cells induced secretion of effector cytokines (IFNγ, TNFα, IL-2, and IL-6) and cytotoxic mediators (Granzyme A and Granzyme B). In contrast, no cytokine production was detected when iCas9.B7-H3 CAR T cells were co-cultured with Jurkat (B7-H3-negative) cells (Fig. S1B, Fig. 2F), confirming antigen-specific activation. The anti-tumor efficacy of iCas9.B7-H3 CAR T cells was evaluated using patient-derived organotypic spheroids (PDOTS) from patients with intrahepatic and hilar extrahepatic cholangiocarcinoma (ECC). ICC and ECC spheroids were cultured with either iCas9.B7-H3 CAR T cells or CD19 CAR T cells. iCas9.B7-H3 CAR T cells effectively infiltrated and eradicated tumor spheroids over 5 days at an E:T ratio of 1:1 compared to CD19 CAR T cells, which did not show cytotoxicity or tumor cell clearance (Fig. 2G). To further characterize the PDOT platform, immunofluorescence staining for CD3, CD33, and CD45 confirmed the presence of T cells, myeloid cells, and broader leukocyte populations within the spheroid cultures (Fig. S4). Together, these findings underscore the target-specific and potent anti-tumor activity of iCas9.B7-H3 CAR T cells against B7-H3-expressing ICC and ECC *in vitro*.

### Systemic iCas9.B7-H3 CAR T cells show dose-dependent anti-tumor activity *in vivo*

**Fig. 3:**
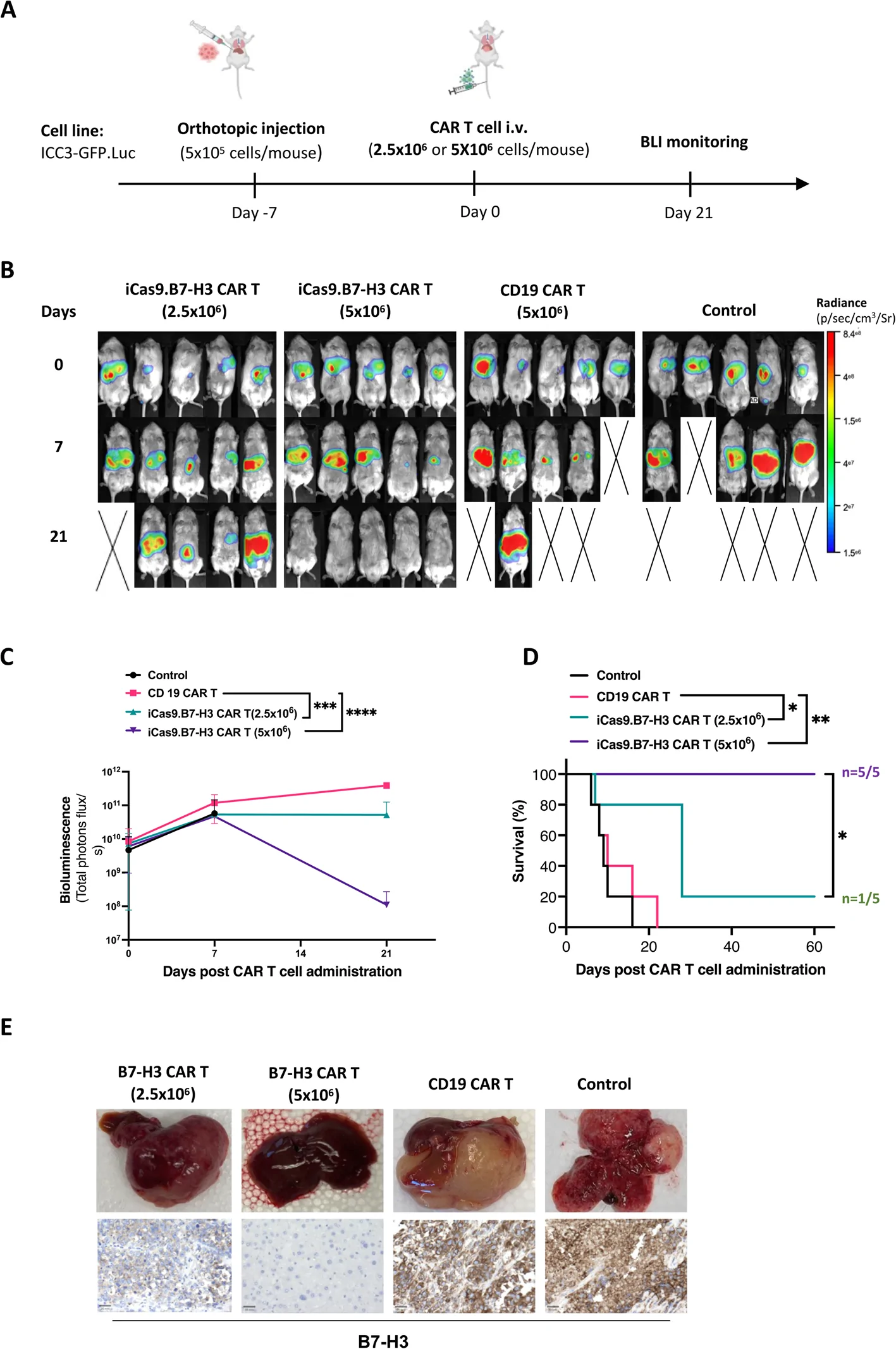
iCas9.B7-H3 CAR T cells demonstrate dose-dependent antitumor activity in an orthotopic ICC model. **A** Schematic representation of the orthotopic ICC xenograft model established using ICC3.GFP-Luc cells and subsequent systemic administration of CAR T cells. **B** Representative bioluminescence imaging (BLI) of tumor burden in mice treated as indicated (*n* = 5 mice/group). “X” denotes mice that died of disease prior to the indicated time point. **C** Quantification of total photon flux over time following CAR T cell administration, demonstrating dose-dependent tumor control (*n* = 5 mice/group). **D** Kaplan–Meier survival analysis of treated mice (*n* = 5 mice/group). **E** Representative gross and histopathologic assessment of tumor burden across treatment groups. Gross images of harvested livers are shown alongside corresponding immunohistochemistry (IHC) staining of formalin-fixed paraffin-embedded sections using anti–human B7-H3 antibody (scale bar, 20 μm). Statistical analysis: Two-way ANOVA with Geisser–Greenhouse correction (**C**) and log-rank (Mantel–Cox) test (**D**). *P ≤ 0.05; **P ≤ 0.01; ***P ≤ 0.001; ****P ≤ 0.0001

In CAR T cell therapy, dose escalation enhances treatment efficacy until a threshold is achieved [42]. To assess the optimal *in vivo* dose of iCas9.B7-H3 CAR T cells, we established an orthotopic ICC model by grafting ICC3 cells transduced with GFP and luciferase (ICC3-GFP.Luc) into the liver of NOD/SCID/γc⁻/⁻ (NSG) mice (Fig. 3A). After confirming the presence of ICC tumors via bioluminescence imaging (BLI) on day 7, mice were randomized into treatment groups (Fig. 3B) and received a systemic infusion of either 2.5 × 10^6^ or 5 × 10^6^ iCas9.B7-H3 CAR T cells. The administration of 5 × 10^6^ iCas9.B7-H3 CAR T cells resulted in the complete eradication of ICC tumors by day 21, with mice remaining tumor-free until the study endpoint at day 60. In contrast, the lower dose (2.5 × 10^6^) did not achieve complete tumor elimination, but significantly delayed tumor progression and prolonged survival in a subset of mice (Fig. 3C-D). No residual ICC was identified macroscopically or by IHC in mice treated with the higher dose (Fig. 3E). These findings demonstrate a dose-dependent therapeutic effect, with higher CAR T-cell doses achieving complete tumor eradication and 100% survival, whereas lower doses provide partial tumor control with an overall survival of 30 days post-CAR T treatment (Fig. 3D).

### iCas9.B7-H3 CAR T cells demonstrate a sustained response to primary ICC tumors and tumor rechallenge

**Fig. 4:**
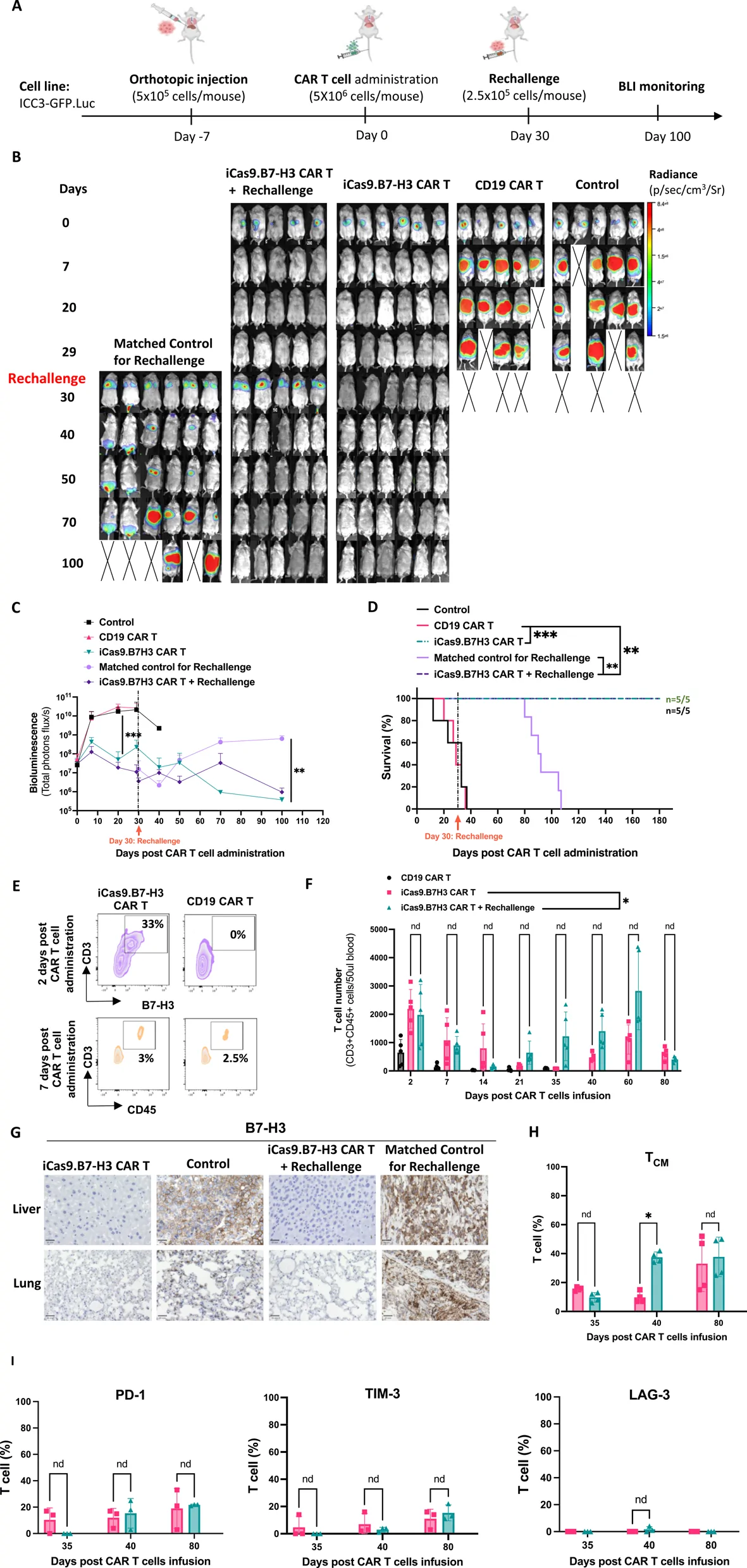
iCas9.B7-H3 CAR T cells mediate sustained antitumor activity and response to tumor rechallenge. **A** Schematic representation of the orthotopic ICC xenograft model (ICC3.GFP-Luc) and CAR T cell administration followed by tumor rechallenge. **B** Representative bioluminescence imaging (BLI) of tumor burden in mice treated as indicated (*n* = 5–6 mice/group). “X” denotes mice that died of disease prior to the indicated time point. **C** Quantification of total photon flux over time following CAR T cell treatment (*n* = 5–6 mice/group). The dashed vertical line indicates the time point of tumor rechallenge (day 30). **D** Kaplan–Meier survival analysis of treated mice (*n* = 5–6 mice/group). The dashed vertical line indicates the time point of tumor rechallenge (day 30), from which the survival of the time-matched control group is introduced and measured. **E** Representative flow cytometry plots of circulating B7-H3 and CD19 CAR T cells in peripheral blood at days 2 and 7 post-infusion. At day 2, CAR T cells were identified by CD3 expression and B7-H3 CAR detection via binding of recombinant B7-H3 protein. At day 7, CAR T cells were identified as CD3⁺CD45⁺ cells. **F** Quantification of circulating T cells (CD3⁺CD45⁺) in peripheral blood (50 μL) of tumor-bearing mice (*n* = 5 mice/group). **G** Representative immunohistochemistry (IHC) staining of formalin-fixed paraffin-embedded liver, lung, and kidney sections using anti–human B7-H3 and anti-CD3 antibodies (scale bar, 20 μm). **H** Frequency of central memory T cells (TCM; CD3⁺CD45⁺CD62L⁺CD45RO⁻) in peripheral blood at days 35, 40, and 80 post–CAR T cell infusion (*n* = 4 biological replicates/group). **I** Frequency of exhaustion-associated markers (PD-1, TIM-3, LAG-3) in circulating CAR T cells (CD3⁺CD45⁺) at days 35, 40, and 80 post-infusion (*n* = 3 biological replicates/group). Statistical analysis: Two-way ANOVA with Geisser–Greenhouse correction and Šídák multiple-comparisons test (F), multiple unpaired t-tests (C, H, I), and log-rank (Mantel–Cox) test (D). *P ≤ 0.05; **P ≤ 0.01; ***P ≤ 0.001

Tumors generally develop resistance to CAR T therapy; thus, we investigated the persistence of iCas9.B7-H3 CAR T cells in our orthotopic ICC model (Fig. 4A). Tumor burden was monitored longitudinally starting from the day of CAR T-cell infusion (defined as day 0). iCas9.B7-H3 CAR T cells not only achieved complete tumor eradication but also demonstrated sustained anti-tumor activity up to 180 days. (Fig. 4B-D). iCas9.B7-H3 CAR T cells were detected in peripheral blood for up to 80 days after infusion (Fig. 4E-F). To evaluate the prolonged functional capacity of circulating iCas9.B7-H3 CAR T cells, we rechallenged mice with tumor cells via tail vein injection of ICC3-GFP.Luc cells at the indicated time point (dashed line in Fig. 4C-D). A matched control group of tumor-free mice that received neither CAR T-cell therapy nor tumor cells was included to confirm successful tumor establishment following tail vein injection of ICC3-GFP.Luc cells. After 180 days, all mice in the original iCas9.B7-H3 CAR T cell treated group, as well as mice with ICC tumor cells via tail vein inoculation, survived without evidence of tumor recurrence, as determined by BLI (Fig. 4B) and macroscopic and IHC assessment of B7-H3 expression in the liver and lung (Fig. 4G). Circulating iCas9.B7-H3 CAR T cells were detected by flow cytometry in all mice before tumor rechallenge on day 21. Following rechallenge, circulating CAR T-cell levels increased, with a peak observed at day 5 post-treatment (Fig. 4F). Although an overall group effect was observed over time, differences in circulating CAR T-cell counts at individual time points did not reach statistical significance after correction for multiple comparisons. In contrast, the CD45RO-CD62L + T memory stem cell population showed a significant increase on day 10 post-rechallenge, indicating a phenotypic shift toward a memory-like state (Fig. 4H). No significant differences in the expression of T-cell exhaustion markers were noted before or after tumor rechallenge (F ig. 4I). To confirm target persistence, B7-H3 expression was verified by IHC in stomachs and intestines collected from mice in both the orthotopic (control) and systemic injection (Matched control for rechallenge) groups (Fig. S5A). To further evaluate potential off-tumor persistence and tissue toxicity, major organs including liver, lung, kidney, stomach, and intestine were examined histologically at the experimental endpoint (day 180 post–CAR T infusion). Hematoxylin–eosin staining demonstrated preserved tissue architecture without evidence of inflammatory injury. Immunohistochemical staining for CD3 revealed rare T cells, indicating minimal CAR T-cell accumulation following tumor clearance (Fig. S5B).

### Locoregional administration of iCas9.B7-H3 CAR T cells effectively eliminates orthotopic ICC xenografts

**Fig. 5:**
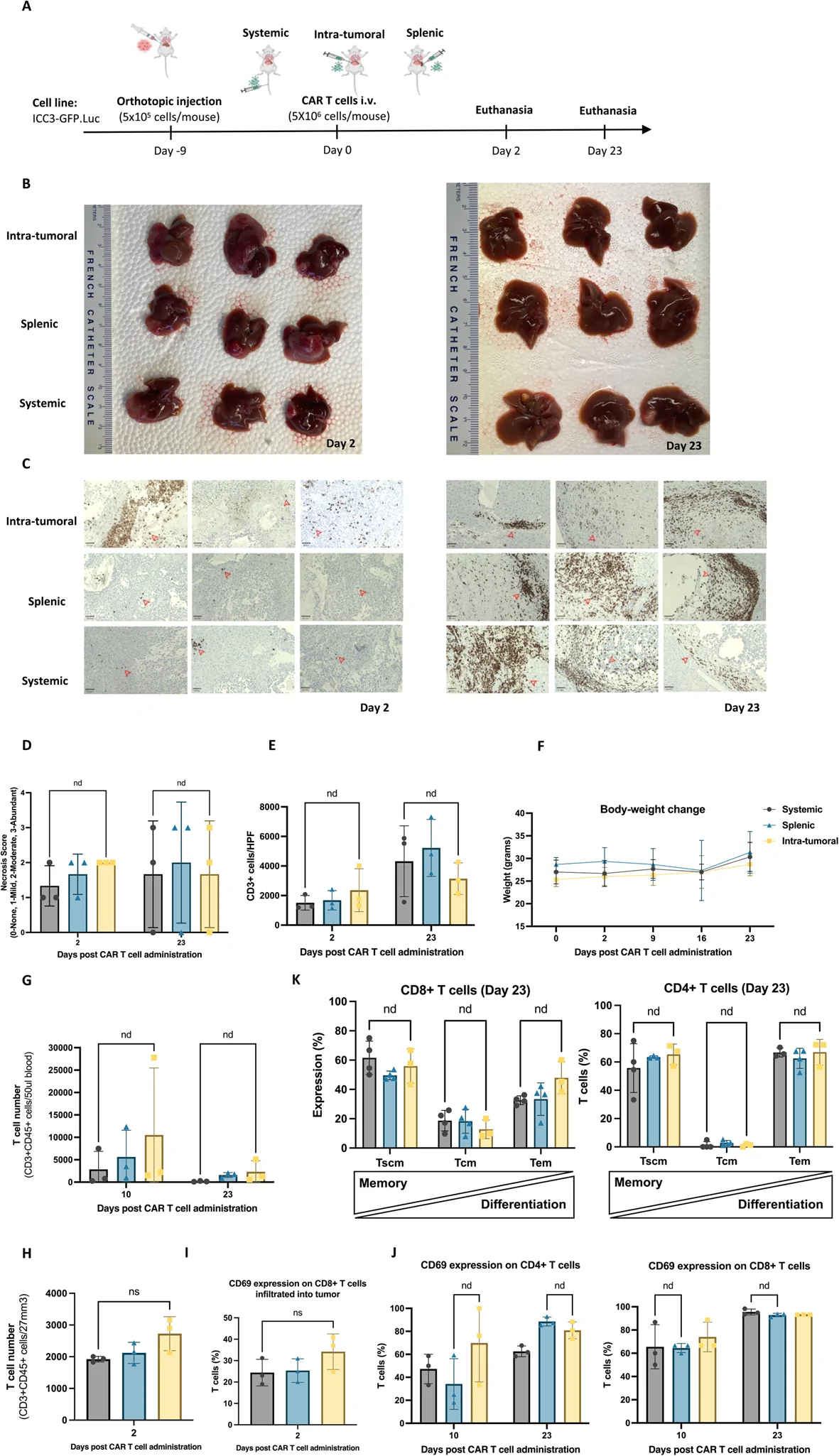
Locoregional administration of iCas9.B7-H3 CAR T cells reduces primary ICC tumor burden. **A** Schematic representation of the orthotopic ICC xenograft model (ICC3.GFP-Luc) and CAR T cell administration routes. **B** Representative gross images of livers from each treatment group. **C** Representative immunohistochemistry (IHC) staining of liver sections (anti-human CD3). CD3⁺ T cells are indicated by red arrows (scale bar, 20 μm). **D** Quantification of tumor necrosis, categorized as mild (1–33%), moderate (34–66%), or abundant (> 67%). Necrosis was scored as 0 (none), 1 (mild), 2 (moderate), or 3 (abundant), with higher scores reflecting increased tumor necrosis. **E** Quantification of CD3⁺ T-cell infiltration across tumor compartments, including tumor periphery, intra-tumoral nests, and infiltrating regions. ImageJ was used to assess T-cell density across treatment groups. **F** Changes in mouse body weight following treatment. **G** Quantification of circulating T cells (CD3⁺CD45⁺) in peripheral blood (50 μL) of tumor-bearing mice (*n* = 3 mice/group). **H** Quantification of tumor-infiltrating T cells (CD3⁺CD45⁺) in primary tumor tissue (27 mm^3^) at day 2 post-treatment (*n* = 3 mice/group). **I** Frequency of activated CD8⁺ T cells (CD69⁺) in primary tumor tissue at day 2 post-treatment (*n* = 3 mice/group). **J** Frequency of activated CD4⁺ and CD8⁺ T cells (CD69⁺) in peripheral blood at days 10 and 23 post-treatment (*n* = 3 mice/group). **K** Phenotypic analysis of CD4⁺ and CD8⁺ T cells in peripheral blood at day 23 post-treatment (*n* = 3 mice/group). Statistical analysis: Unpaired t-test

CAR T therapy is often limited by systemic toxicity or insufficient infiltration into the tumor. Liver-restricted tumors offer the possibility for locoregional treatment via hepatic artery infusion to minimize toxicity while increasing CAR T cell delivery to target lesions [43]. To assess the optimal route for iCas9.B7-H3 CAR T cell administration *in vivo*, we employed our orthotopic ICC model by grafting ICC3-GFP.Luc cells into the livers of NSG mice (Fig. 5A). After confirming tumor growth on day 9 by BLI imaging, mice were randomized into treatment groups and received iCas9.B7-H3 CAR T cells via splenic injection, intra-tumoral injection, or tail vein injection. We treated mice with CAR T-cells at day 9 post tumor cell inoculation and assessed T-cell infiltration. Two days post-treatment, a subset of mice was euthanized. While gross tumor sizes were similar across all groups, IHC revealed a trend toward higher T-cell infiltration in the intra-tumoral group (Fig. 5B-C). By day 23, CAR T cells induced marked tumor regression across all groups, with no significant difference between systemic (tail vein) or locoregional (splenic and intra-tumoral) administration routes. (Fig. 5B-D). At the conclusion of the survival experiment, T-cell infiltration into the liver was observed across all treatment groups, with variable distribution between administration routes (Fig. 5C, E). Notably, no significant toxicity was observed, and body weight remained stable across all groups (Fig. 5F). On day 10, T-cell counts in peripheral blood were highest in the intra-tumoral group, although this difference was not statistically significant (Fig. 5G), A trend toward increased intratumoral T-cell presence was observed at day 2 (Fig. 5H) while CD69 expression, a marker of T-cell activation, was higher in the intra-tumoral group in both tumor tissue (day 2) and peripheral blood (day 10); however, these differences were not statistically significant (F ig. 5I-J). By day 23, there was an increased presence of T memory stem cells and effector memory T cells across all treatment groups, indicating a durable immune response (Fig. 5K). Collectively, these findings demonstrate that both systemic and locoregional administration routes achieve comparable tumor control, with no statistically significant differences in T-cell infiltration or activation between groups.

## Discussion

The limited efficacy of current therapeutic options for intrahepatic cholangiocarcinoma (ICC) and the high expression of the immunomodulatory antigen B7 homolog 3 (B7-H3) on tumor cells and components of the tumor microenvironment (TME) support its evaluation as a therapeutic target [26, 32–34]. In this study, we provide integrated orthogonal evidence—including flow cytometry of patient specimens, tissue microarray analysis, and public transcriptomic and proteomic datasets—demonstrating tumor-associated enrichment of B7-H3 with minimal expression in normal hepatobiliary tissues, supporting a potential therapeutic window for B7-H3–directed CAR T-cell therapy. Although not statistically significant, the trend toward reduced overall survival in tumors with higher B7-H3 expression suggests a potential association with immune evasion and aggressive tumor behavior, consistent with prior reports [26, 31].

Consistent with these expression data, our functional studies demonstrated that iCas9.B7-H3 CAR T cells exhibit potent and antigen-specific cytotoxic activity against ICC cell lines and patient-derived tumor spheroids, with robust cytokine production and proliferation upon antigen engagement. These findings support effective CAR T-cell activation independent of HLA class I presentation, a key limitation in ICC due to frequent defects in antigen presentation machinery. While prior CAR T-cell strategies targeting EGFR and HER2 have shown variable efficacy in biliary tract cancers [44–46], our data suggest that B7-H3–directed CAR T cells may overcome some of these limitations through consistent antigen expression and tumor targeting. Our data suggest that B7-H3–directed CAR T cells may overcome some of these limitations through consistent antigen expression and tumor targeting. In a recent study, PTG-T16R-B7-H3-CAR-T cells demonstrated durable tumor control in humanized mouse models [47]; however, residual tumor burden at study endpoint suggests that further optimization of CAR design or combination strategies may still be required.

Current ICC tumor antigens exhibit limited and heterogeneous expression across tumors, contributing to suboptimal responses to CAR T cell immunotherapy [48]. In contrast, we observed uniformly high B7-H3 expression across multiple ICC cell lines and patient samples, including cancer-initiating cell populations, supporting its relevance as a therapeutic target capable of addressing both differentiated tumor cells and tumor-initiating compartments. Given the role of cancer-initiating cells in recurrence and metastasis [49, 50], targeting B7-H3 may contribute to improved disease control and reduced relapse risk.

*In vivo*, systemic administration of iCas9.B7-H3 CAR T cells resulted in complete tumor eradication in an orthotopic ICC model and demonstrated durable antitumor activity, including protection following tumor rechallenge with newly implanted tumor cells, confirming the development of functional immunologic memory. These findings highlight the capacity of this platform to achieve sustained tumor control. The observed efficacy may in part reflect the specificity of the 376.96-derived scFv; however, it is important to note that the use of immunodeficient NSG models and CAR T cells derived from healthy donor PBMCs may overestimate therapeutic performance relative to clinical settings.

Locoregional chemotherapy delivery via the hepatic artery has been successfully applied in liver malignancies to enhance tumor exposure while limiting systemic toxicity [51]. Motivated by this clinical paradigm, we evaluated alternative locoregional CAR T-cell delivery approaches, including splenic and intra-tumoral administration. Due to technical limitations of hepatic artery injection in the murine model, splenic injection was used to approximate portal venous delivery. While intra-tumoral injection has shown promise in other solid tumors [52–54], concerns remain regarding distribution and persistence. In our study, no statistically significant differences in T-cell infiltration, activation, or tumor regression were observed across delivery routes, although early time-point trends suggested increased intratumoral T-cell presence following intra-tumoral administration. Importantly, overall tumor control was comparable between systemic and locoregional delivery approaches. These findings indicate that locoregional administration is feasible in this model but do not support a clear advantage over systemic delivery, and further evaluation in immunocompetent or clinically relevant models is required.

Our study builds upon prior work demonstrating significant preclinical efficacy of B7-H3 CAR T cells across multiple solid tumors, including those currently undergoing clinical investigation [37, 55–57]. To enhance safety, our construct incorporates an inducible caspase-9 (iCas9) suicide switch, a clinically validated strategy that enables rapid elimination of CAR T cells in the event of severe toxicity. We and others have previously demonstrated efficient *in vivo* ablation of iCas9-engineered CAR T cells following administration of a dimerizing agent [41]. While comprehensive cytokine profiling and toxicity assessment were beyond the scope of this study, the inclusion of this safety mechanism provides an important translational safeguard, particularly given known risks such as cytokine release syndrome (CRS) and immune effector cell–associated neurotoxicity syndrome (ICANS) [58].

Consistent with this, histologic evaluation of major organs demonstrated preserved tissue architecture without evidence of inflammatory injury, and CAR T cells were minimally detectable at late time points following tumor clearance, suggesting limited long-term off-tumor persistence in this model. Nevertheless, these findings should be interpreted with caution given the immunodeficient nature of the NSG model, and further safety evaluation in humanized or immunocompetent systems will be required.

Despite these promising results, several limitations should be acknowledged. First, the NSG xenograft model lacks key components of the human immune system, including regulatory T cells, tumor-associated macrophages, and other immunosuppressive elements of the TME, which may influence CAR T-cell trafficking, persistence, and function. While PDOT models partially preserve tumor architecture and stromal components [38] they do not fully recapitulate *in vivo* immune complexity. Second, CAR T cells generated from healthy donor PBMCs may not reflect the functional impairments observed in patient-derived T cells, including increased exhaustion and reduced memory phenotypes [55]. Additionally, tumor recurrence remains a major challenge due to antigen heterogeneity, immune escape, and TME-mediated suppression [59, 60]. Future studies should therefore focus on evaluating this platform in more clinically relevant models, including humanized or immunocompetent systems, and on developing combinatorial strategies to overcome TME-mediated resistance. Potential approaches include CAR T-cell engineering to enhance trafficking and persistence, as well as combination with chemotherapy, radiotherapy, or metabolic modulators such as disulfiram/copper [55, 57, 61]. 

## Conclusions

In conclusion, our findings demonstrate that iCas9.B7-H3 CAR T cells exhibit potent and durable antitumor activity in preclinical models of intrahepatic cholangiocarcinoma. These results support B7-H3 as a promising therapeutic target and highlight the potential of incorporating a safety-switch–enabled CAR T-cell platform for the treatment of ICC. Further studies are warranted to address the limitations of current preclinical models and to evaluate the safety, efficacy, and translational applicability of this approach in clinically relevant settings.

## Supplementary Information

Supplementary Material 1

## Data Availability

The data supporting the findings of this study are available from the corresponding author upon reasonable request, subject to approval by the Massachusetts General Hospital and Cedars-Sinai Medical Center Institutional Animal Care and Use Committees (IACUC).

## Abbreviations

CAR: Chimeric Antigen Receptor
ICC: Intrahepatic Cholangiocarcinoma
B7-H3: B7 Homolog 3 (CD276)
iCas9: Inducible Caspase-9
PDOTs: Patient-Derived Organotypic Tumor Spheroids
PBMCs: Peripheral Blood Mononuclear Cells
CICs: Cancer-Initiating Cells
TME: Tumor Microenvironment
NSG: NOD scid gamma (immunodeficient mouse model)
IL-2: Interleukin-2
IL-6: Interleukin-6
IL-7: Interleukin-7
IL-15: Interleukin-15
IFN-γ: Interferon gamma
TNF-α: Tumor Necrosis Factor alpha
E:T: Effector-to-Target Ratio
IV: Intravenous
IP: Intraperitoneal
PBS: Phosphate-Buffered Saline
FACS: Fluorescence-Activated Cell Sorting
BLI: Bioluminescence Imaging
IVIS: *In Vivo* Imaging System
IHC: Immunohistochemistry
TMA: Tissue Microarray
qPCR: Quantitative Polymerase Chain Reaction
TCR: T Cell Receptor
TCM: Central Memory T Cells
ICANS: Immune Effector Cell–Associated Neurotoxicity Syndrome
CRS: Cytokine Release Syndrome
IRB: Institutional Review Board
IACUC: Institutional Animal Care and Use Committee
MGH: Massachusetts General Hospital
CSMC: Cedars-Sinai Medical Center
