# Correction: B7-H3 CAR T cells eradicate intrahepatic cholangiocarcinoma and induce durable response

**DOI:** 10.1186/s13046-026-03825-0

**Published:** 2026-09-19

**Authors:** Shahrzad Arya, Marco Ventin, Giulia Cattaneo, Martina Nebbia, Seyed Amir Sanatkar, Cristina Martin, Jingyu Jia, Bangjin Kim, Cedric A. Bailey, Nabeel M. Bardeesy, David T. Ting, Vikram Deshpande, Xinhui Wang, Sandra Ryeom, Soldano Ferrone, Cristina R. Ferrone

**Affiliations:** 1 Division of Gastrointestinal and Surgical Oncology, Department of Surgery, Massachusetts General Hospital, Harvard Medical School, Boston, MA USA; 2 Department of Surgery, Cedars Sinai Medical Center, Los Angeles, CA USA; 3 Herbert Irving Comprehensive Cancer Center, Department of Surgery, Columbia University Irving Medical Center, New York, NY USA; 4 Department of Pathology, Cedars Sinai Medical Center, Los Angeles, CA USA; 5 MassGeneral Cancer Center, Massachusetts General Hospital, Harvard Medical School, Boston, MA USA; 6 Department of Pathology, Beth Israel Deaconess Medical Center, Boston, MA USA

**Correction: J Exp Clin Cancer Res 45, 131 (2026)**

**https://doi.org/10.1186/s13046-026-03723-5**

Following the official publication of the original article [1], the authors identified that the figures processed for the final publication were not the accepted versions. The figure captions were published correctly and required no changes. In addition, the published Supplementary Material inadvertently contained internal comments, which have now been removed. The original article and Supplementary Material have been updated accordingly.

The corrections do not compromise the validity of the conclusions and the overall content of the article. The original article [1] has been corrected.
