# Supplementary material for: B7-H3 CAR T cells eradicate intrahepatic cholangiocarcinoma and induce durable response
Source: J Exp Clin Cancer Res. 2026 Jun 4;45:131. doi: 10.1186/s13046-026-03723-5 (PMC13255452; doi:10.1186/s13046-026-03723-5)
Supplement: Supplementary file 1 — Supplementary Material 1 [file 13046_2026_3723_MOESM1_ESM.docx]

**Table S1. Quantitative B7-H3 expression in matched normal and tumor specimens from patients with cholangiocarcinoma.**

B7-H3 expression was quantified by flow cytometry and reported as the percentage of B7-H3–positive cells in matched normal liver, normal bile duct, primary tumor, and metastatic ICC samples. Each row represents an individual patient specimen. Matched normal tissues demonstrate minimal B7-H3 expression, whereas primary and metastatic tumor samples exhibit higher expression levels. Missing values are indicated by “–”.

**Figure S1. Confirmation of target antigen specificity in ICC and control cell lines.**

**(A)** Representative flow cytometry histograms demonstrating CD19 expression in ICC cell lines stained with anti–human CD19 mAb (1 μg/mL), confirming minimal or absent CD19 expression. **(B)** Representative flow cytometry histograms demonstrating B7-H3 expression in the Jurkat cell line stained with anti–human B7-H3 mAb (376.96, 1 μg/mL), confirming minimal or absent B7-H3 expression.

**Figure S2. Public dataset analyses support tumor-associated B7-H3 expression in cholangiocarcinoma.**

**(A)** B7-H3 mRNA expression in cholangiocarcinoma tumors (n = 36) compared with normal hepatobiliary tissues (n = 9) from the TCGA-CHOL dataset analyzed using GEPIA2. **(B)** Kaplan–Meier overall survival analysis of patients in the TCGA-CHOL cohort stratified by B7-H3 expression (high vs low groups defined by median expression). **(C)** B7-H3 transcript expression across normal hepatobiliary cell populations obtained from the Human Protein Atlas dataset, including cholangiocytes, hepatocytes, endothelial cells, stellate cells, and macrophages. **(D)** Distribution of B7-H3 expression across cholangiocarcinoma cell lines derived from publicly available transcriptomic datasets. Statistical analysis: One-way ANOVA for differential expression **(A)**; survival analysis performed using Cox proportional hazards model with significance assessed by log-rank test **(B)**. Hazard ratio (HR) = 1.4; *P ≤ 0.05.

**Figure S3. iCas9.B7-H3 CAR T cells maintain transduction efficiency and *in vitro* functionality.**
**(A)** Representative flow cytometry histograms showing transduction efficiency of B7-H3 CAR and iCas9.B7-H3 CAR T cells using FITC-labeled B7-H3 protein (n = 3 independent experiments from PBMCs derived from three healthy donors). **(B)** Bar plots showing the percentage of ICC cell viability following co-culture with iCas9.B7-H3 or B7-H3 CAR T cells at different effector-to-target (E:T) ratios, assessed by MTT assay after 3 days (n = 3 independent experiments). **(C)** Proliferation of iCas9.B7-H3 or B7-H3 CAR T cells following administration of AP1903 (10 nM) in co-culture assays with CAR T viability assessed by cell counting. Statistical comparisons were performed using paired t-tests. ****p ≤ 0.0001.

**Figure S4. Cellular composition of patient-derived organotypic tumor spheroids (PDOTs) and infiltration of B7-H3 CAR T cells.
(A)** Flow cytometric analysis of PDOT cellular composition showing tumor cells (EpCAM⁺), T cells (CD3⁺), and myeloid cells (CD33⁺), presented as the percentage of total cells. **(B)** Representative flow cytometry plots illustrating gating of EpCAM⁺ tumor cells, CD3⁺ T cells, and CD33⁺ myeloid cells within PDOTs. **(C)** Quantification of T-cell populations in untreated PDOTs and PDOTs co-cultured with B7-H3 CAR T cells, including T cells (CD3⁺CAR⁻) and CAR T cells (CD3⁺CAR⁺). **(D)** Representative flow cytometry plots, demonstrating T cells (CD3⁺CAR⁻) and CAR T cell (CD3⁺CAR⁺) populations.

**Figure S5. Gastrointestinal metastases in control mice and histologic evaluation of potential off-tumor CAR T cell persistence.**
**(A)** Immunohistochemical (IHC) analysis of gastrointestinal metastases in control mice. Stomach and intestinal tissues were collected following orthotopic tumor implantation (control) and systemic tumor rechallenge (matched control for rechallenge) with ICC3-GFP.Luc cells. FFPE sections were stained with rabbit anti-human B7-H3 antibody (1:400), demonstrating B7-H3 expression in metastatic lesions (scale bar, 20 μm). **(B)** Histologic evaluation of potential off-tumor CAR T cell persistence and tissue toxicity. Major organs, including liver, lung, kidney, stomach, and intestine, were examined at the experimental endpoint (day 180 post–CAR T infusion). Hematoxylin–eosin and anti-human CD3 staining demonstrated preserved tissue architecture without evidence of inflammatory injury or detectable T-cell infiltration. IHC staining with rabbit anti-human CD3 antibody (1:400) showed no detectable CD3⁺ T cells in the examined tissues (scale bar, 20 μm).
