# Supplementary material for: B7-H3 CAR T cells eradicate intrahepatic cholangiocarcinoma and induce durable response
Source: J Exp Clin Cancer Res. 2026 Jun 4;45:131. doi: 10.1186/s13046-026-03723-5 (PMC13255452; doi:10.1186/s13046-026-03723-5)
Supplement: Supplementary file 2 [file 13046_2026_3723_MOESM1_ESM.pdf]

Sample ID	Matched Normal Liver (%)	Normal Bile Duct (%)	Primary Tumor (%)	ICC Metastasis (%)
**1**	5.5	–	64.8	–
**2**	4.2	12.6	61.9	–
**3**	4.0	12.0	63.2	–
**4**	1.1	16.7	90.0	–
**5**	0.7	0.8	77.2	–
**6**	2.4	–	93.0	–
**7**	1.9	–	97.0	–
**8**	2.9	–	60.0	–
**9**	5.0	–	67.8	–
**10**	7.6	–	43.9	–
**11**	5.0	–	55.2	–
**12**	6.2	–	75.6	–
**13**	–	–	–	99.6

Table S1. Quantitative B7-H3 expression in matched normal and tumor specimens from patients with cholangiocarcinoma. B7-H3 expression was quantified by flow cytometry and reported as the percentage of B7-H3–positive cells in matched normal liver, normal bile duct, primary tumor, and metastatic ICC samples. Each row represents an individual patient specimen. Matched normal tissues demonstrate minimal B7-H3 expression, whereas primary and metastatic tumor samples exhibit higher expression levels. Missing values are indicated by "–".

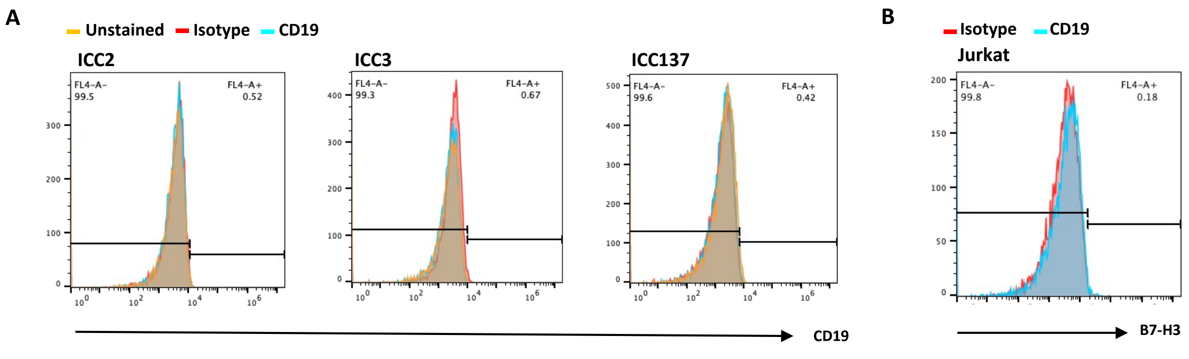

Figure S1. Confirmation of target antigen specificity in ICC and control cell lines. (A) Representative flow cytometry histograms demonstrating CD19 expression in ICC cell lines stained with anti–human CD19 mAb (1 µg/mL), confirming minimal or absent CD19 expression. **(B)** Representative flow cytometry histograms demonstrating B7-H3 expression in the Jurkat cell line stained with anti–human B7-H3 mAb (376.96, 1 µg/mL), confirming minimal or absent B7-H3 expression.

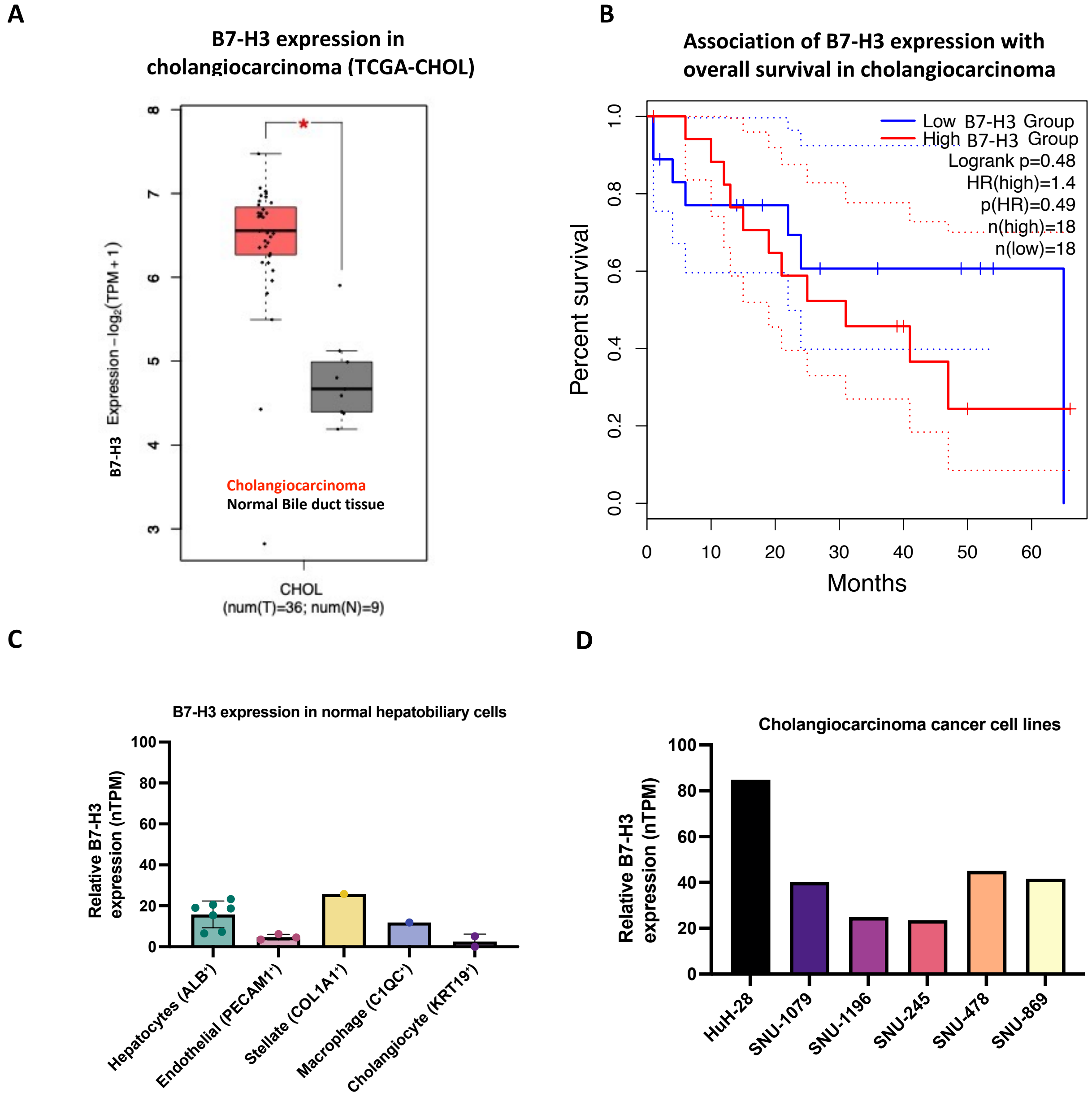

Figure S2. Public dataset analyses support tumor-associated B7-H3 expression in cholangiocarcinoma. **(A)** B7-H3 mRNA expression in cholangiocarcinoma tumors (n = 36) compared with normal hepatobiliary tissues (n = 9) from the TCGA-CHOL dataset analyzed using GEPIA2. **(B)** Kaplan–Meier overall survival analysis of patients in the TCGA-CHOL cohort stratified by B7-H3 expression (high vs low groups defined by median expression). **(C)** B7-H3 transcript expression across normal hepatobiliary cell populations obtained from the Human Protein Atlas dataset, including cholangiocytes, hepatocytes, endothelial cells, stellate cells, and macrophages. **(D)** Distribution of B7-H3 expression across cholangiocarcinoma cell lines derived from publicly available transcriptomic datasets. Statistical analysis: One-way ANOVA for differential expression **(A)**; survival analysis performed using Cox proportional hazards model with significance assessed by log-rank test **(B)**. Hazard ratio (HR) = 1.4; *P ≤ 0.05.

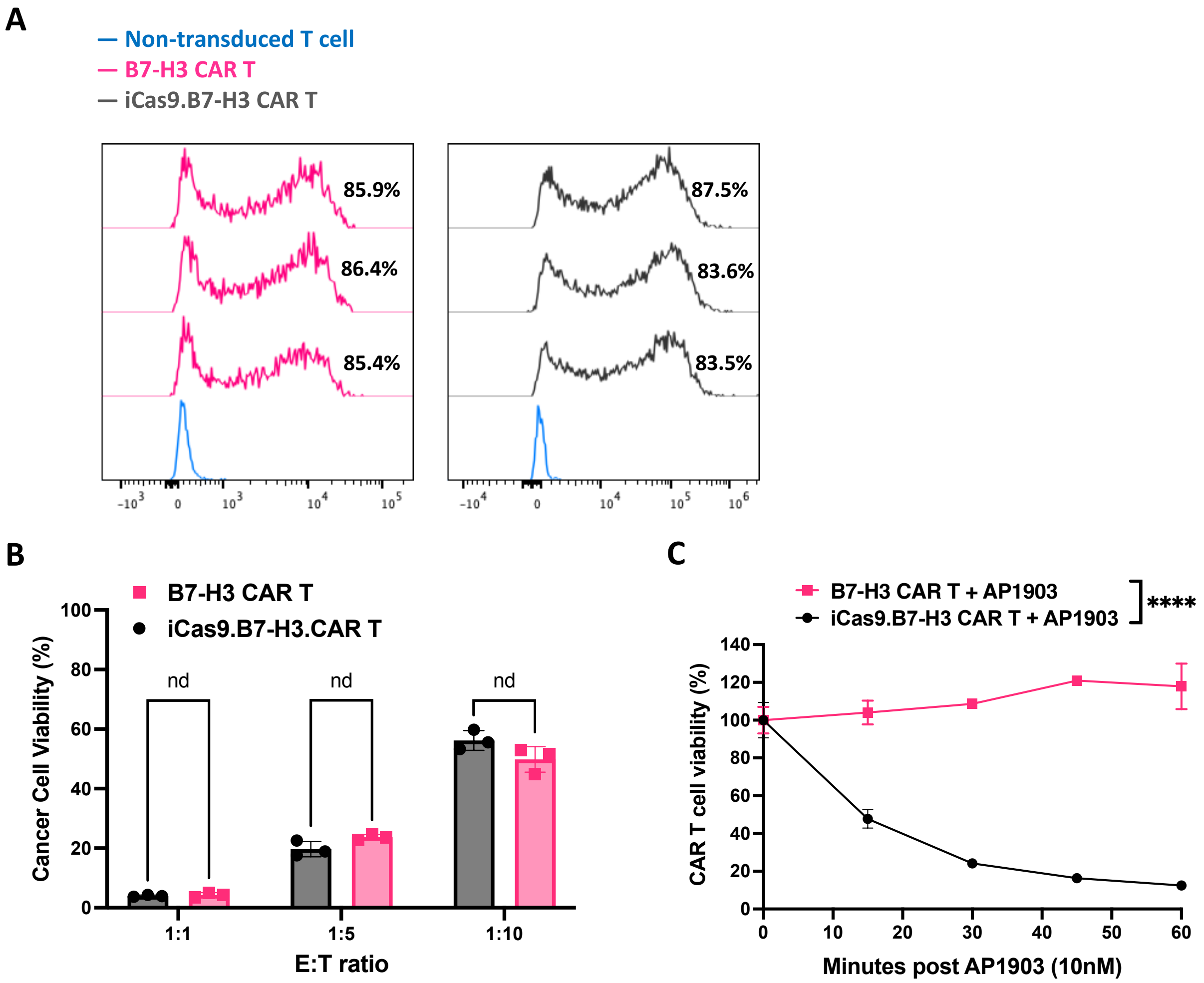

Figure S3. iCas9.B7-H3 CAR T cells maintain transduction efficiency and *in vitro* functionality. (A) Representative flow cytometry histograms showing transduction efficiency of B7-H3 CAR and iCas9.B7-H3 CAR T cells using FITC-labeled B7-H3 protein (n = 3 independent experiments from PBMCs derived from three healthy donors). **(B)** Bar plots showing the percentage of ICC cell viability following co-culture with iCas9.B7-H3 or B7-H3 CAR T cells at different effector-to-target (E:T) ratios, assessed by MTT assay after 3 days (n = 3 independent experiments). **(C)** Proliferation of iCas9.B7-H3 or B7-H3 CAR T cells following administration of AP1903 (10 nM) in co-culture assays with CAR T viability assessed by cell counting. Statistical comparisons were performed using paired t-tests. ****$p \leq 0.0001$.

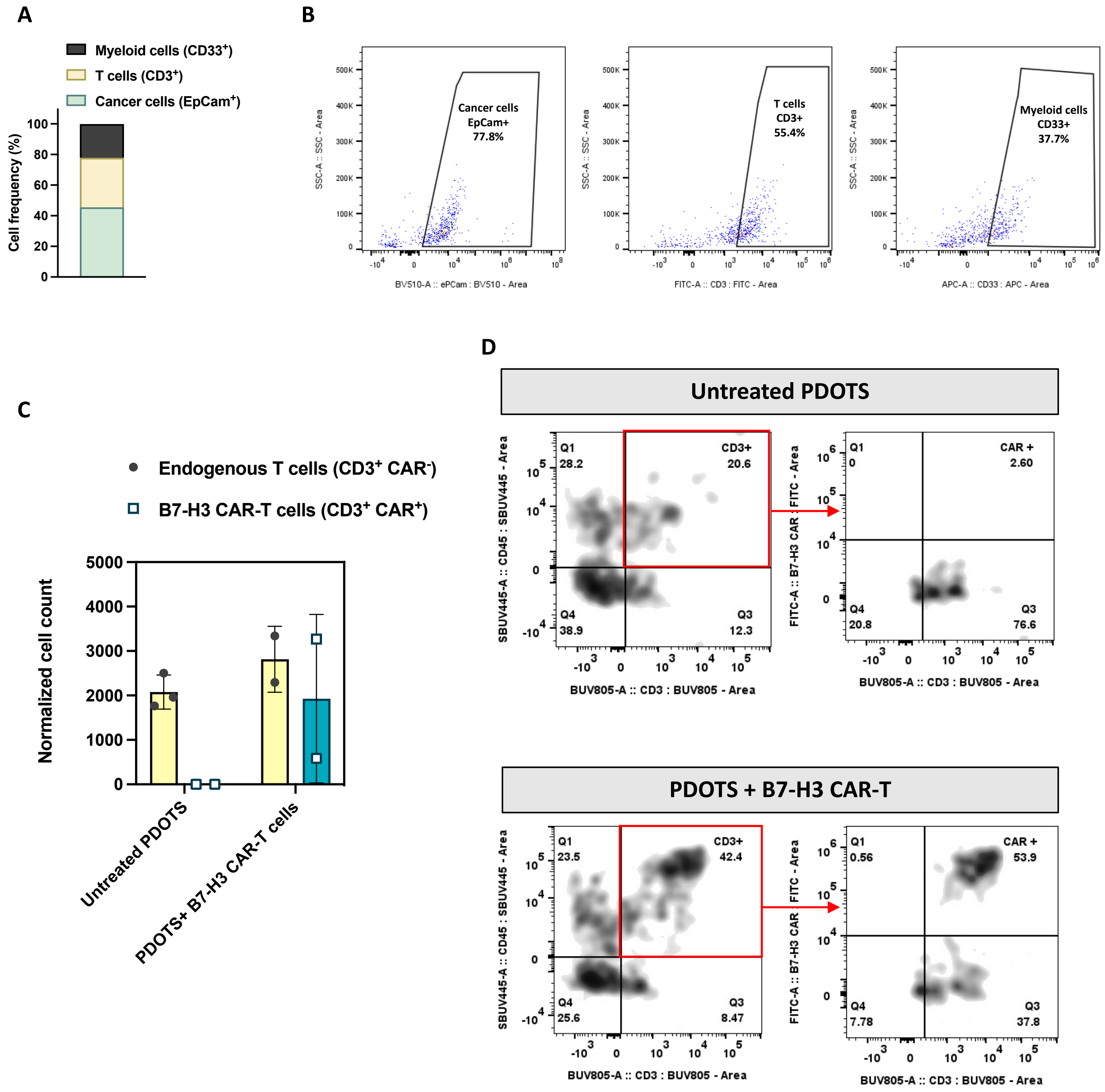

Figure S4. Cellular composition of patient-derived organotypic tumor spheroids (PDOTs) and infiltration of B7-H3 CAR T cells. (A) Flow cytometric analysis of PDOT cellular composition showing tumor cells (EpCAM$^+$), T cells (CD3$^+$), and myeloid cells (CD33$^+$), presented as the percentage of total cells. **(B)** Representative flow cytometry plots illustrating gating of EpCAM$^+$ tumor cells, CD3$^+$ T cells, and CD33$^+$ myeloid cells within PDOTs. **(C)** Quantification of T-cell populations in untreated PDOTs and PDOTs co-cultured with B7-H3 CAR T cells, including T cells (CD3$^+$CAR$^-$) and CAR T cells (CD3$^+$CAR$^+$). **(D)** Representative flow cytometry plots, demonstrating T cells (CD3$^+$CAR$^-$) and CAR T cell (CD3$^+$CAR$^+$) populations.

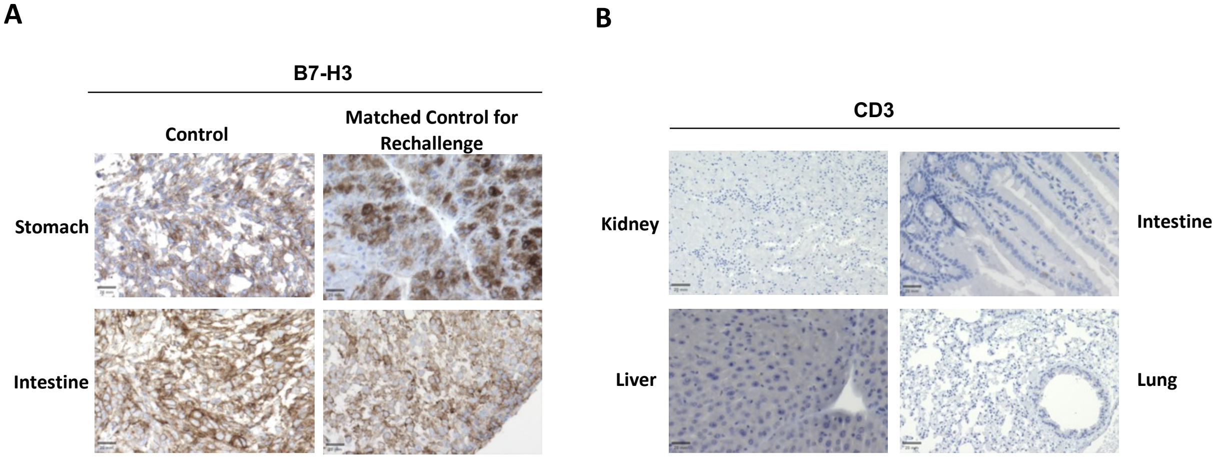

Figure S5. Gastrointestinal metastases in control mice and histologic evaluation of potential off-tumor CAR T cell persistence. (A) Immunohistochemical (IHC) analysis of gastrointestinal metastases in control mice. Stomach and intestinal tissues were collected following orthotopic tumor implantation (control) and systemic tumor rechallenge (matched control for rechallenge) with ICC3-GFP.Luc cells. FFPE sections were stained with rabbit anti-human B7-H3 antibody (1:400), demonstrating B7-H3 expression in metastatic lesions (scale bar, 20 μm). **(B)** Histologic evaluation of potential off-tumor CAR T cell persistence and tissue toxicity. Major organs, including liver, lung, kidney, stomach, and intestine, were examined at the experimental endpoint (day 180 post–CAR T infusion). Hematoxylin–eosin and anti-human CD3 staining demonstrated preserved tissue architecture without evidence of inflammatory injury or detectable T-cell infiltration. IHC staining with rabbit anti-human CD3 antibody (1:400) showed no detectable $CD3^+$ T cells in the examined tissues (scale bar, 20 μm).
